# Microcirculation-on-a-Chip: A Microfluidic Platform for Assaying Blood- and Lymphatic-Vessel Permeability

**DOI:** 10.1371/journal.pone.0137301

**Published:** 2015-09-02

**Authors:** Miwa Sato, Naoki Sasaki, Manabu Ato, Satoshi Hirakawa, Kiichi Sato, Kae Sato

**Affiliations:** 1 Department of Chemical and Biological Sciences, Faculty of Science, Japan Women’s University, Bunkyo, Tokyo, Japan; 2 Department of Immunology, National Institute of Infectious Diseases, Shinjuku-ku, Tokyo, Japan; 3 Department of Dermatology at Hamamatsu University School of Medicine, Hamamatsu city, Shizuoka, Japan; 4 Division of Molecular Science, School of Science and Technology, Gunma University, Kiryu, Gunma, Japan; University of Illinois at Chicago, UNITED STATES

## Abstract

We developed a microfluidic model of microcirculation containing both blood and lymphatic vessels for examining vascular permeability. The designed microfluidic device harbors upper and lower channels that are partly aligned and are separated by a porous membrane, and on this membrane, blood vascular endothelial cells (BECs) and lymphatic endothelial cells (LECs) were cocultured back-to-back. At cell-cell junctions of both BECs and LECs, claudin-5 and VE-cadherin were detected. The permeability coefficient measured here was lower than the value reported for isolated mammalian venules. Moreover, our results showed that the flow culture established in the device promoted the formation of endothelial cell-cell junctions, and that treatment with histamine, an inflammation-promoting substance, induced changes in the localization of tight and adherens junction-associated proteins and an increase in vascular permeability in the microdevice. These findings indicated that both BECs and LECs appeared to retain their functions in the microfluidic coculture platform. Using this microcirculation device, the vascular damage induced by habu snake venom was successfully assayed, and the assay time was reduced from 24 h to 30 min. This is the first report of a microcirculation model in which BECs and LECs were cocultured. Because the micromodel includes lymphatic vessels in addition to blood vessels, the model can be used to evaluate both vascular permeability and lymphatic return rate.

## Introduction

Microcirculation plays a crucial role in the supply of oxygen and nutrients from the blood to extravascular tissues. The microcirculation system consists of blood and lymphatic vascular capillaries and the interstitium, and functional disorders of the microcirculation system are strongly related to, for example, inflammatory responses, swelling, and tumor. When an injury occurs in a body, the inflammatory response, which is induced by released inflammation-promoting agents such as histamine or cytokines, affects the microvascular system [1]. Inflammation is characterized by several familiar signs such as redness, swelling, heat, and pain. The best-characterized responses of the microcirculation system to inflammation include impaired vasomotor function, reduced capillary perfusion, adhesion of leukocytes and platelets, activation of the coagulation cascade, enhanced thrombosis, increased vascular permeability, and an increase in the rate of proliferation of blood and lymphatic vessels. Lymphedema is a disorder of the lymphatic vascular system that is characterized by impaired lymphatic return and swelling of the extremities [2]. Several intractable diseases are also related to microcirculation disorders. Most tumor vasculatures are immature and are recognized to exhibit highly enhanced vascular permeability. Conversely, previous studies have also revealed pericyte coverage of human tumor vasculature, which reduces vascular permeability and the effects of cancer-treatment drugs [3]. Therefore, in vitro models of microcirculation suitable for studying its physiological mechanism are required for developing treatments for intractable diseases.

To date, the physiological mechanism underlying microcirculation has been investigated using numerous experiments involving animals and cultured cells. In experiments conducted using small animals, blood and lymph flows were measured by observing the movements of erythrocytes and leukocytes in the vessels [4]. Vascular permeability was also measured using surgery-based observation of the leakage of macromolecular fluorescent probes such as labeled dextran or albumin after a defined period of circulation [5–7].

Conversely, blood flow in the human body has been measured using noninvasive techniques. In vivo vascular imaging has been performed using imaging methods such as computed tomography, magnetic resonance imaging, optical coherence tomography, and confocal laser-scanning microscopy, and using Doppler ultrasound imaging, a non-optical method [8]. However, these methods do not provide sufficient resolution for revealing the microvascular structure, because blood vascular and lymphatic capillaries feature diameters of approximately 5–10 μm [9] and 10–60 μm [10], respectively. The major drawbacks of the optical approaches include low resolution and low penetration depth. Moreover, lymphatic vessels have been imaged to a lesser extent than blood vessels because the density of lymphatic vessels is lower than that of blood vessels.

Compared to animal experiments, experiments on cultured normal human microvascular endothelial cells or normal human lymphatic microvascular endothelial cells are conducted more widely because the behavior of these cells can be monitored in detail. The effects of vascular or lymph flow have also been examined using endothelial cell monolayers cultured on glass slides or petri dishes under various flow rates of culture media [11,12]. The permeation functions of endothelial cell monolayers cultured on transwell permeable supports were previously analyzed using fluorescence-labeled dextran tracers or by measuring transepithelial electrical resistance in order to confirm the formation of a permeability barrier [13–16]. However, using these methods, physiological functions were mimicked insufficiently because the systems used were highly simplified and did not closely resemble the corresponding systems in the human body, and thus the results obtained were distinct from those of animal testing.

Recent advances in microfluidic technologies have opened the door for creating in vitro cell-culture formats that are more realistic than those used previously. The size scale of the microchannels in microfluidic devices is close to that of the microvascular system and these microchannel-based cell cultures can be used as perfusion cultures, and, consequently, microvascular models have been realized. In a vascular model, endothelial cells have been stimulated under flow conditions mimicking vascular flow [17,18], and microfluidic devices have now been developed for various applications, such as for permeation assays performed using a fluorescence-labeled dextran tracer [19–25]; for studies on angiogenesis performed using extracellular matrix (ECM) hydrogels [26–28]; and for mimicking the blood-brain barrier, a selective barrier that is unique to the central nervous system [29–31]. By contrast, only 2 reports have been published on a lymphatic vascular model, in which the permeation assay was performed with lymphatic flow effects [32,33]. The microcirculation system’s primary function is the maintenance of interstitial fluid homeostasis, based on permeation from the blood vessels and subsequent absorption by lymphatic vessels. Therefore, it is necessary for the microcirculation model to represent the functions of lymphatic as well as blood vessels.

In this study, our aim was to develop a microcirculation model featuring both blood and lymphatic vessels for the purpose of examining vascular permeability. We focused the interaction between blood vascular endothelial cells (BECs) and lymphatic endothelial cells (LECs), and in the newly developed device, the flow conditions mimicked vascular and lymphatic flow and molecules moved from blood vessels to lymphatic vessels through an ECM hydrogel.

## Materials and Methods

### Device fabrication

**Fig 1:**
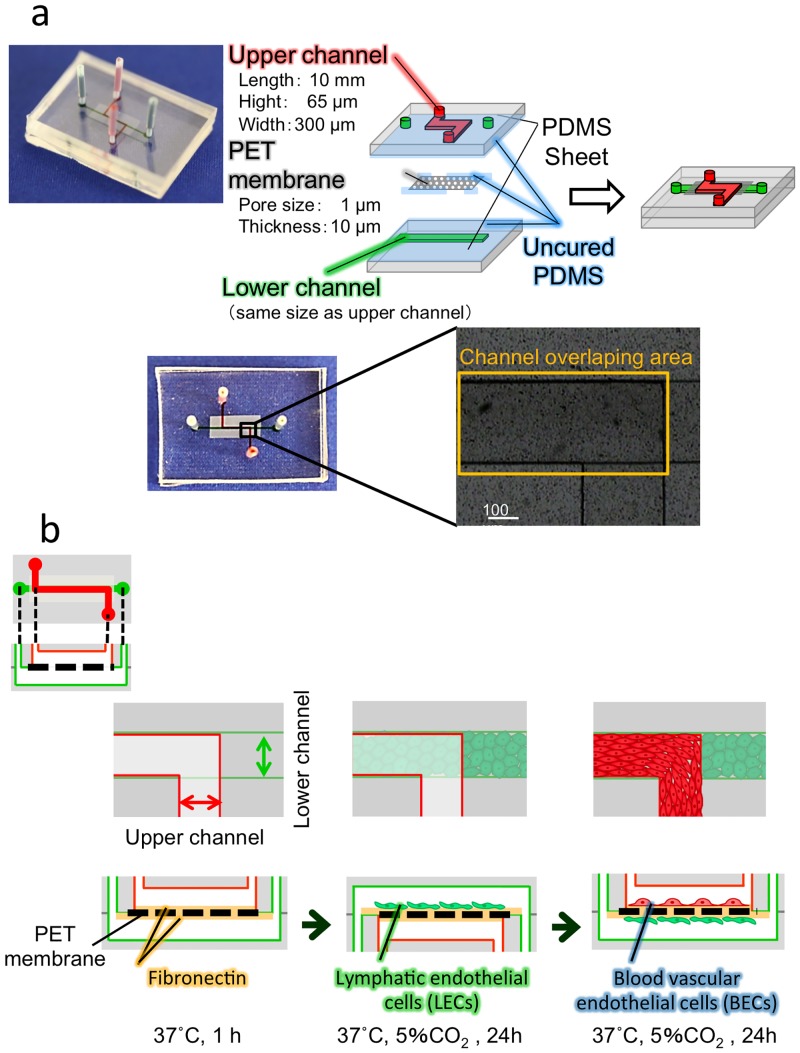
Experimental design. (a) Schematic of a membrane-integrated microfluidic device. (b) Experimental procedure used for coculturing BECs (cells in red) and LECs (cells in green) on opposite sides of the membrane in the microfluidic device.

Porous polyethylene terephthalate (PET) membranes (pore size, 1.0 μm) were cut from a cell-culture insert (353102; Becton Dickinson, Franklin Lakes, NJ, USA) to a size of 3 × 8 mm. Microfluidic devices harboring porous membranes were fabricated using the method described previously [34], which was based on the procedures reported by Chueh *et al*. [35]. Poly(dimethylsiloxane) (PDMS, Silpot 184; Dow Corning, Midland, MI, USA) was used for preparing substrates (23 × 18 mm) featuring microchannel patterns (300 μm wide, 65 μm deep, 10 mm long). A 1.0-μm-pore membrane was integrated into a microfluidic device as follows. First, a PDMS-hexane mixture of weight ratio 10:1:33 (prepolymer:curing agent:hexane), referred to as PDMS mortar, was spin-coated on a glass slide at 2000 rpm for 30 s and then placed for 10 min at room temperature to allow the hexane to evaporate. Next, a PDMS substrate bearing an upper-channel was stamped onto the glass slide coated with the PDMS mortar, and 2 min later, it was peeled off. A small amount of PDMS mortar was placed on the edges of the porous membrane and allowed to sit for 10 min, after which the membrane was placed on a pattern of the upper microchannel of the substrate (Fig 1a). Subsequently, the PDMS substrate featuring a lower-microchannel pattern was placed on the membrane. The PDMS-membrane composite was degassed for 30 min, and then baked at 100°C for 1 h under pressure applied using a 1-kg weight.

A schematic of the microfluidic device is presented in Fig 1a. The upper and lower channels are partly overlapped and are separated by a membrane that allows the fluorescent tracer to permeate from the upper channel to the lower channel.

In certain experiments, we used a single-channel microdevice lacking the porous membrane. This microdevice contains a straight channel (300 μm × 65 μm × 20 mm), and to fabricate it, a PDMS sheet featuring the microchannel structure was bonded with a glass substrate after both surfaces were treated with oxygen plasma.

Each end of a microchannel was connected to a polytetrafluoroethylene (PTFE) tube (0.46 mm id, 0.92 mm od, 8 mm long; Nichias, Tokyo, Japan). One end of the PTFE tube was connected to a 1-mL syringe (Terumo, Tokyo, Japan) through a bubble trap, a PFA capillary (0.1 × 0.3 × 750 mm), and a 23G needle (Nonaka Rikaki, Tokyo, Japan). The bubble trap was fabricated in accordance with published details [36] and was composed of 2 TYGON tubes of dimensions 0.79 mm id and 2.38 mm od and one tube of dimensions 2 mm id and 4 mm od (Saint-Gobain K.K., Tokyo, Japan). The other PTFE tube was connected to a TYGON tube.

### Microfluidic cell culture

Normal human dermal microvascular endothelial cells (HMVEC-d) and normal human lymphatic microvascular endothelial cells (HMVEC-dLy) (both from Lonza, Basel, Switzerland) were used as BECs and LECs, respectively. Cells were grown in EBM-2 supplemented with EGM-2 BulletKit (Lonza). Cells between passages 3 and 8 were used for all experiments. Once cells reached confluence, the medium in cell culture flasks was aspirated, and the cells were rinsed with 5 mL of PBS(-) (TAKARA BIO, Shiga, Japan) and then treated with 1 mL of TrypLE Express (Life Technologies, Carlsbad, CA, USA). After the cells detached from the surface of the flask, 2 mL of fresh medium containing 10% FBS was added, and the obtained cell suspension was transferred to a 15-mL conical tube. The tube was centrifuged at 1200 rpm for 3 min and the supernatant was aspirated, and the cells were then resuspended in culture medium at the required concentration.

The microfluidic cell culture is illustrated in Fig 1b. The porous membrane was coated with fibronectin by incubating it with 0.1 mg/mL fibronectin at 4°C for 16 h and then at 37°C for 1 h. After washing with fresh medium, 2 μL of the HMVEC-dLy suspension (1.5 × 10^7^ cells/mL) was introduced into the lower microchannel by using a 50-μL microsyringe (Hamilton, Reno, NV, USA) equipped with a 25G or 27G needle (Terumo). The device was inverted to allow HMVEC-dLy to attach to the backside of the membrane, wrapped with a wet lint-free wiper (BEMCOT M-1, Asahi Kasei, Tokyo, Japan) to prevent desiccation, and incubated under static conditions for 6 h in a 5% CO_2_ incubator at 37°C. After incubation, the cells were cultured under pulsating-flow, continuous-flow, or static conditions. After culturing for 18 h, the microchannel was turned upside down, and the same amount of the HMVEC-d suspension was introduced into the upper channel. After incubation for 6 h to allow cells to attach to the upper side of the membrane, the cells in both channels were cultured for 18 h under a 1-μL/h pulsating flow generated using a miniaturized infusion pump (SMP101-L, Primetech, Tokyo, Japan) or a 1-μL/h continuous flow (pressure drops, Δ*P* = 4 dyn/cm^2^; shear stresses, *τ* = 0.01 dyn/cm^2^) generated using a syringe pump (Model 210 or 230 KD Scientific, Holliston, MA, USA), or under static conditions (S1 Fig). The pressure drops Δ*P* [37] and shear stresses *τ* [38] were calculated by the following equation:

$$\Delta P=\frac{12\mu LQ}{wh^{3}}$$ (1)

$$\tau =\frac{6\mu Q}{wh^{2}}$$ (2)

where *Q*: flow rate, *μ*: viscosity, *L*: length of the channel, *w*: width of the channel and *h*: height of the channel.

### Cell-viability assay

Cell-viability assays were performed using 2 fluorescent dyes (LIVE/DEAD Viability/Cytotoxicity assay kit, Life Technologies). Cells were reacted with 2 μM Calcein AM and 4 μM ethidium homodimer in the culture media for 30 min at 37°C and under 5% CO_2_, and then rinsed with PBS(+).

### Immunostaining

Cells cultured in microchannels were immunostained for the lymphatic marker podoplanin [39], and for VE-cadherin/CD144 [40] and claudin-5 [41], which are key components of endothelial adherens and tight junctions, respectively. The reagents and buffer were introduced into the microchannel manually and incubated under a stopped-flow condition.

To stain for podoplanin, cells cultured for 24 h were washed with PBS(+), fixed with 4% paraformaldehyde (PFA) at 4°C for 2 min, rinsed with PBS(+), blocked with PBS containing 1% BSA (Wako Pure Chemical Industries, Osaka, Japan) for 30 min at room temperature, rinsed with PBS(+) thrice for 2 min each, and then reacted with mouse D2-40 monoclonal antibody against podoplanin (Nichirei, Tokyo, Japan) for 16 h at 4°C. The cells were then rinsed with PBS(+) thrice for 2 min each, reacted with 10 μg/mL Alexa Fluor 488-conjugated goat anti-mouse IgG antibody (Life Technologies) in 1% BSA for 1 h at room temperature, and rinsed with PBS(+) thrice for 2 min each.

To stain for claudin-5, a rabbit polyclonal anti-claudin-5 antibody (Abcam, Cambridge, UK) and Alexa Fluor 555-conjugated goat anti-rabbit IgG antibody were used as primary and secondary antibodies, respectively. In the case of VE-cadherin/CD144 staining, a mouse monoclonal anti-human VE-cadherin/CD144 antibody (R&D Systems, Minneapolis, MN, USA) and Alexa Fluor 488-conjugated goat anti-mouse IgG antibody (Life Technologies) were used as primary and secondary antibodies, respectively. Cells cultured for 24 h were washed with PBS(+) twice for 2 min each, fixed with 4% PFA at 4°C for 15 min, rinsed with PBS(+) twice for 2 min each, treated with 0.3% Triton X-100 in PBS(+) for 5 min, blocked with PBS containing 1% BSA (Wako Pure Chemical Industries) for 30 min at room temperature, rinsed with PBS(+) twice for 2 min each, and then reacted with 10 μg/mL primary antibody in 1% BSA for 16 h at 4°C. The cells were then rinsed with PBS(+) twice for 2 min each, reacted with 10 μg/mL secondary antibody in 1% BSA (Life Technologies) for 30 min at room temperature, and rinsed with PBS(+) twice for 2 min each. Cell nuclei were stained with 10 μg/mL Hoechst33342 (Life Technologies).

### Detection of histamine binding

In the histamine-stimulation assay, BODIPY-FL-histamine (Life Technologies) was used for detecting the specific binding of histamine on the cell surface. Cells cultured for 24 h under a 6-μL/h continuous-flow condition were incubated for 2 min with either 1 μM BODIPY-FL-histamine or 1 μM BODIPY-FL-BSA (negative control; Life Technologies), and then washed with PBS(+).

### Ca^2+^ imaging

Ca^2+^ imaging was performed using Calcium Kit-Fura2 (Dojindo, Kumamoto Japan). All solutions were pumped into the microchannel at 6 μL/h by using the syringe pump. Cells cultured for 24 h under a 6-μL/h continuous-flow condition (generated using the syringe pump) were incubated with a Fura2-AM-containing loading buffer (recording medium, 0.04% Pluronic F-127, 0.04% Cremophor EL, 1.25 mM Probenecid, and 5 mg/L Fura2-AM) for 1 h at 37°C. Next, the Fura2-AM-containing buffer was replaced with recording medium containing 1.25 mM Probenecid. Cells were treated with 1 or 100 mM histamine in recording medium.

### Permeation tests

The inflammatory mediator histamine and habu snake venom were used as test compounds that exhibit vascular leakage activity. Permeation tests were conducted by introducing culture media containing the test compound, 100 mM histamine-2HCl (Kanto Chemical Co., Inc., Tokyo, Japan) or 29 the minimum haemorrhagic dose (MHD)/mL habu snake venom hemorrhagic factor-II (HR-2) (The National Institute of Infectious Diseases, Tokyo, Japan), and fluorescent tracers, 10 μM tetramethylrhodamine isothiocyanate (TRITC)-dextran (FW = 40,000, Sigma-Aldrich, St. Louis, MO, USA) and Lucifer Yellow (LY, FW = 457, Sigma-Aldrich), into the upper channel (BEC channel) by using the syringe pump. The flow rate at the inlet of the upper channel was set at 6 μL/h (pressure drops, Δ*P* = 24 dyn/cm^2^; shear stresses, *τ* = 0.07 dyn/cm^2^). In the lower channel, culture media containing the test compound without fluorescent tracers was introduced, and the channel was then not connected with a pump and was maintained under static conditions. Fluorescence intensity was monitored near the bifurcation point of the microchannels in order to estimate the amount of the permeated fluorescent tracer (S2 Fig). The permeability coefficient *P* was calculated from the following equation [42]:

$$P=\frac{\Delta C_{\text{L}}V_{\text{L}}}{C_{\text{U}}A\Delta t}$$ (3)

where *C*_U_ is the initial concentration in the upper channel, Δ*C*_L_ is the concentration change in the lower channel, *V*_L_ is the volume of the lower channel, *A* is the area of membrane that allows fluorescent tracers to permeate from the upper channel to the lower channel, and Δ*t* is the assay time. Lastly, the permeability of BECs or LECs, *P*_*e*_, was calculated from the following equation, where *P*_b_ is the permeability of the device without cells:

$$\frac{1}{P_{\text{e}}}=\frac{1}{P}-\frac{1}{P_{\text{b}}}$$ (4)

After the permeation test, cell viability was evaluated by using data collected from the microdevices in which most of the cells were alive.

### Microscopy

Fluorescence images were obtained using an IX71 Microscope (Olympus, Tokyo, Japan) equipped with a 100-W high-pressure mercury lamp and a cooled CCD camera, ORCA-R2 (Hamamatsu Photonics, Hamamatsu, Japan). For observation of claudin-5, ethidium homodimer, and TRITC-dextran, the dichroic mirror block U-MWIG3 (excitation 530–550 nm, emission > 575 nm) was used. For observation of VE-cadherin, BODIPY-FL-histamine, and LY, another dichroic mirror block, U-MNIBA3 (excitation 470–495 nm, emission 510–550 nm), was used. Stained nuclei were visualized using the dichroic mirror block U-MNUA2 (excitation 360–370 nm, emission 420–460 nm).

For Ca^2+^ imaging, fluorescence was monitored using a microscope equipped with a 75-W Xenon lamp; a cooled CCD camera, Cascade 512F (Photometrics, Tucson, AZ, USA); and a Fura2-specific dichroic mirror block, 79001 (Chroma Technology, Bellow Falls, VT, USA). For time-lapse imaging, Fura2-AM emissions at 510 nm were recorded every 5 s for 5 min by using excitation at 340 and 380 nm, and the emissions corresponded to bound and unbound Ca^2+^ fractions, respectively.

Images were processed using the image analysis software ImageJ 1.45f (National Institutes of Health, MD, USA).

## Results and Discussion

### Coculture of blood vascular and lymphatic endothelial cells in the microdevice

**Fig 2:**
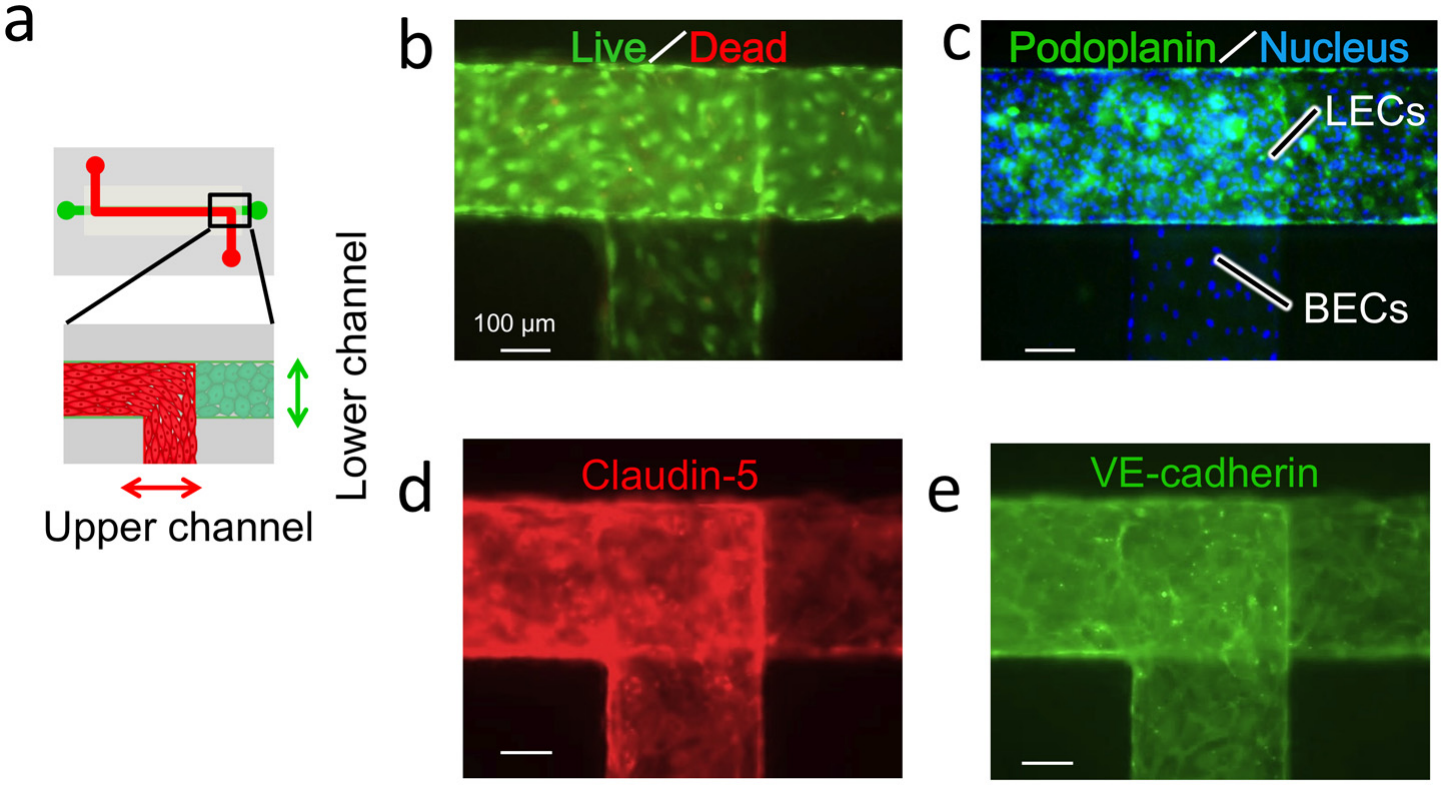
Characterization of BECs and LECs cocultured in the microfluidic device. (a) Schematic of the microfluidic device. The cells in red are BECs and the cells in green are LECs. (b) Live/dead analysis. (c) Detection of the lymphatic endothelium by immunostaining for a lymphatic marker, podoplanin. (d) Detection of an endothelial-specific protein, claudin-5, by immunostaining. (e) Detection of an endothelial-specific adhesion molecule, VE-cadherin, by immunostaining.

Cell-viability analysis and immunostaining for a lymph-specific marker and transmembrane proteins that mediate cell-cell adhesion were performed on cells cultured for 24 h under static conditions in the designed microdevice (Fig 2). Fig 2b shows cell viability in the microdevice, which was measured using the live/dead viability/cytotoxicity assay kit. Most of the LECs and BECs were alive (green). Dead cells were not observed in the microscopic field, which suggests that the occurrence of cell death was below the detection limit.

Fig 2c shows a fluorescence image of cells stained with anti-podoplanin (lymphatic marker) and Hoechst33342 (nuclei). Podoplanin was detected only in the lower channel, which indicates that cells in the lower channel were LECs and those in the upper channel were BECs. Thus, the 2 types of cells were fully separated into the upper and lower channels and did not leak into the other channel. The back-to-back coculture was successful, and the cells grew well on the both sides of the permeable membrane.

Next, we performed immunofluorescence staining for VE-cadherin/CD144 and claudin-5, which are key components of endothelial adherens and tight junctions, respectively, on cells cultured in the microchannel; the results are shown in Fig 2d and 2e. Whereas distinct staining could not by readily observed under the microscope at the back-to-back coculture zone (*i*.*e*., at the overlapping zone), clear immunostaining was observed at the separated channel zones. Claudin-5 and VE-cadherin were expressed at cell-cell junctions in both BECs and LECs. The expression patterns of Claudin-5 and VE-cadherin were similar in both cells, which indicated that both of these types of cells retained their barrier functions required for regulating permeability in the microfluidic coculture.

### Permeability of cells in microfluidic cocultures

**Fig 3:**
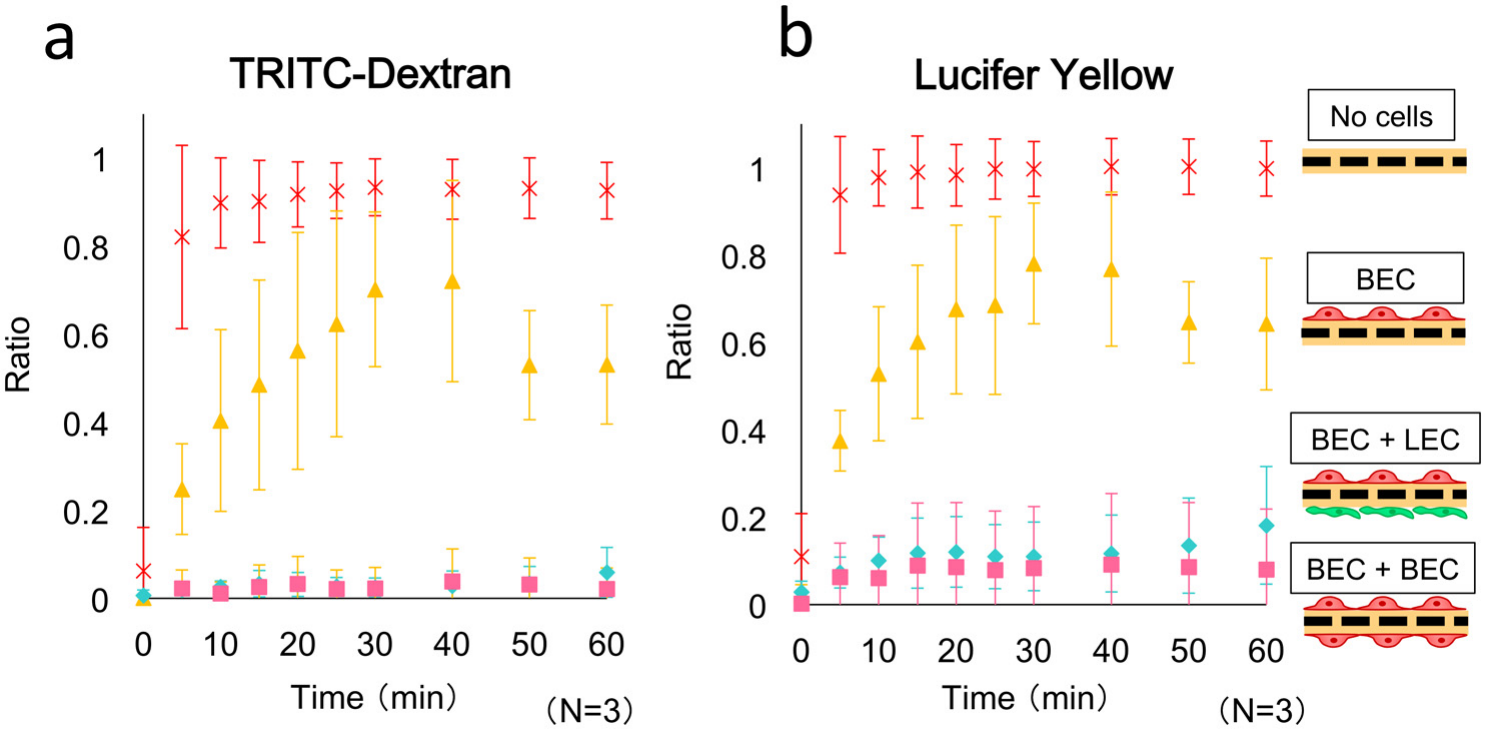
Permeability measured using the microfluidic system lacking or containing BECs and LECs. No cells (x), BEC monolayer (yellow triangles), BEC-LEC coculture (blue diamonds), and BEC-BEC coculture (pink squares). The values are presented as means ± SD. The fluorescent tracers used were (a) TRITC-dextran and (b) Lucifer Yellow. The statistical significance of the differences between the values obtained at 60 min was evaluated using *t* tests: (a) *P* < 0.005, between No cells and BEC monolayer; *P* < 0.001, between BEC monolayer and BEC-LEC coculture; and *P* < 0.32, between BEC-LEC coculture and BEC-BEC coculture. (b) *P* < 0.001, between No cells and BEC monolayer; *P* < 0.001, between BEC monolayer and BEC-LEC coculture; and *P* < 0.45, between BEC-LEC coculture and BEC-BEC coculture. Each data point was obtained from three devices (n = 3).

We analyzed the permeability of the cell layers from the BEC channel to the LEC channel by using TRITC-dextran (FW = 40,000) as a high-molecular-weight fluorescent tracer and LY (FW = 457) as a small-molecule tracer; the tracers were included in the culture media injected into the upper channel by using the syringe pump. Fig 3 shows the results of the permeability test; averages obtained from three experiments are plotted, and the device without any cells was used in the control experiment. In the coculture system, we detected very little leakage of LY and no leakage of TRITC-dextran in the 1-h permeation test. Thus, BECs and LECs appeared to retain their barrier functions due to the formation of endothelial tight junctions.

The permeability of the BEC-LEC coculture was compared with those of the BEC monolayer in the upper channel and the BEC-BEC coculture. The design of the device used in permeation tests was the same as that used in previous assays. BEC-LEC and BEC-BEC cocultures exhibited comparable levels of TRITC-dextran permeation. The calculated permeability coefficient was 0.60 × 10^−6^ cm/s for 40-kDa TRITC-dextran, which was slightly lower than the value reported for isolated mammalian venules (2 × 10^−6^ cm/s for FITC-albumin) [43] and LEC tubes in vitro (1.7 × 10^−6^ cm/s for 10-kDa dextran) [32]. The TRITC-dextran permeation level of the BEC monolayer was higher than the levels measured for both cocultures (16.7 × 10^−6^ cm/s for 40-kDa TRITC-dextran). The value obtained from the BEC monolayer was slightly higher than that reported for BEC tubes in vitro (4.1×10^−6^ cm/s for 70-kDa dextran, 7.0×10^−6^ cm/s for fluorescein (MW = 332) [44], 0.892×10^−6^ cm/s for 70-kDa dextran [45] and 1.55×10^−6^ cm/s for 70-kDa FITC-dextran [46]). In these previous studies, the culture periods were much longer (4 d [45], 6–7 d [46], or 14 d [44]) than the present work (24 h). The lower permeability in these studies might be attributed to stronger cell-cell junction formation owing to longer culture periods.

The results of the LY-permeation tests revealed that the permeation level of the BEC-LEC coculture was slightly higher than that of the BEC-BEC coculture and considerably lower than that of the BEC monolayer. Therefore, we suggest that barrier function, which regulates permeability across cell-cell junctions, increased in this order: BEC monolayer, BEC-LEC coculture, and BEC-BEC coculture.

### Effects of perfusion on the permeability of the endothelial barrier

**Fig 4:**
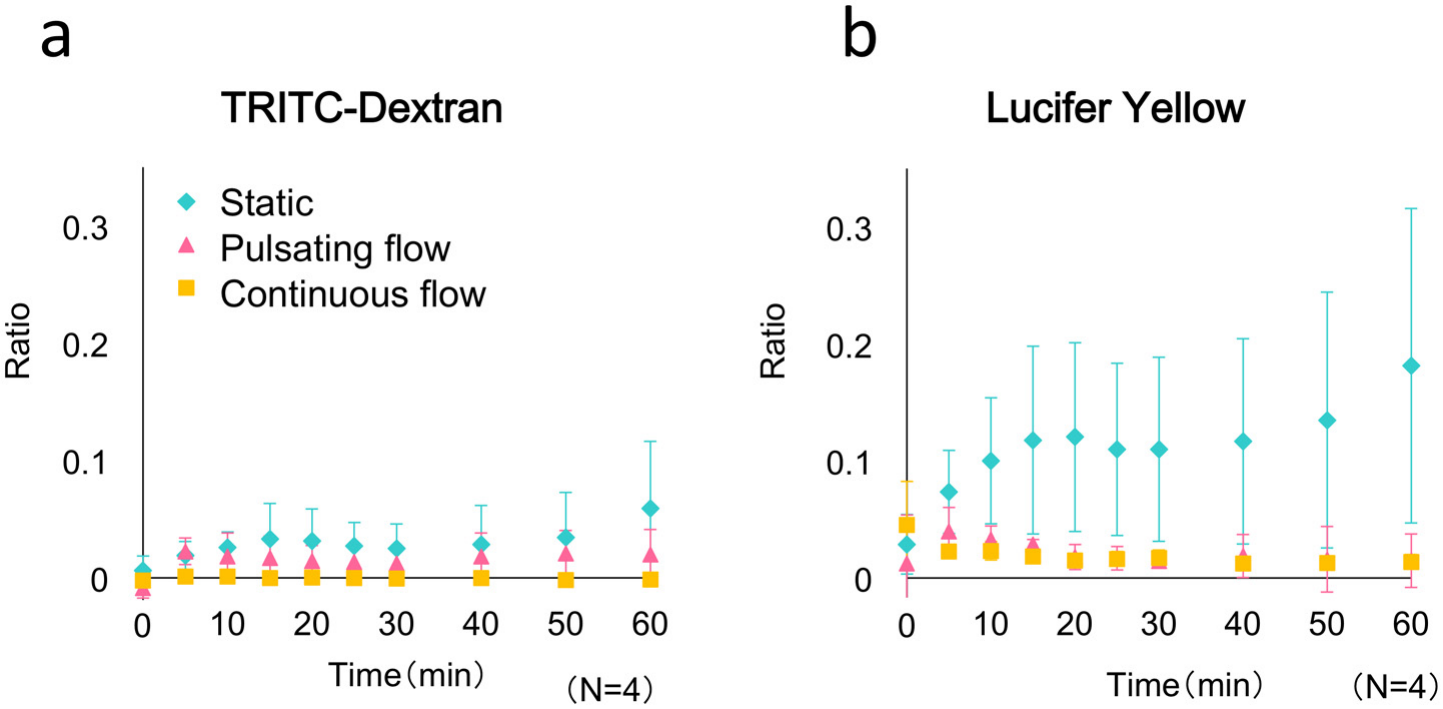
Permeability measured using the microfluidic system containing BECs and LECs cultured under 3 conditions. Permeability tests were performed after cells were cultured for 24 h (BEC-LEC coculture) under a 1-μL/h pulsating-flow condition generated using a miniaturized infusion pump (pink triangles), a 1-μL/h continuous-flow condition generated using a syringe pump (yellow squares), or the static condition (blue diamonds). The values are presented as means ± SD. The fluorescent tracers used were (a) TRITC-dextran and (b) Lucifer Yellow. The significance of differences between the 60-min values was assessed by performing *t* tests: (a) *P* < 0.05, between static condition and continuous-flow condition; and (b) *P* < 0.05, between static condition and both flow conditions. Each data point was obtained from four devices (n = 4).

Permeability tests were conducted using TRITC-dextran and LY on the BEC-LEC bilayer cultured in the microdevice for 24 h under a 1-μL/h pulsating-flow condition generated using the miniaturized infusion pump, a 1-μL/h continuous-flow condition generated using the syringe pump, or the static condition; the results are shown in Fig 4. The cells cultured under the 3 conditions exhibited comparable levels of TRITC-dextran permeation. However, the LY-permeation level of the cells cultured under continuous- and pulsating-flow conditions was lower than that of the static-culture cells. The permeability coefficient measured for LY in the flow cultures was 0.5–1.0 × 10^−6^ cm/s. These results suggest that flow cultures promoted the formation of endothelial cell-cell junctions.

### Reproduction of histamine-induced inflammatory responses in the microdevice

Inflammation-promoting substances such as histamine, thrombin, vascular endothelial growth factor, tumor necrosis factor-alpha, and reactive oxygen species are widely recognized to enhance permeability across endothelial cell-cell junctions [47]. Histamine enhances endothelial permeability through a Ca^2+^-dependent mechanism. The signaling cascade is initiated by the binding of histamine to its receptor on the endothelial surface, which activates G proteins. This process induces intracellular Ca^2+^ signaling and continuously disrupts cell-cell junction structures, which leads to the formation of intercellular gaps [48]. In previous studies, the response to stimulation by inflammation-promoting substances was confirmed using a vascular model [21][29][46][49]. Thus, we also investigated the response to histamine stimulation in our device. To examine the responses of BECs and LECs separately, in the assays described next, a single-channel device lacking the permeable membrane was used instead of the coculture device. The responses to histamine stimulation were investigated by examining the binding of fluorescent-histamine to the cell surface, the elevation of cytosolic Ca^2+^ levels, and the immunofluorescence staining for proteins of endothelial adherens and tight junctions.

**Fig 5:**
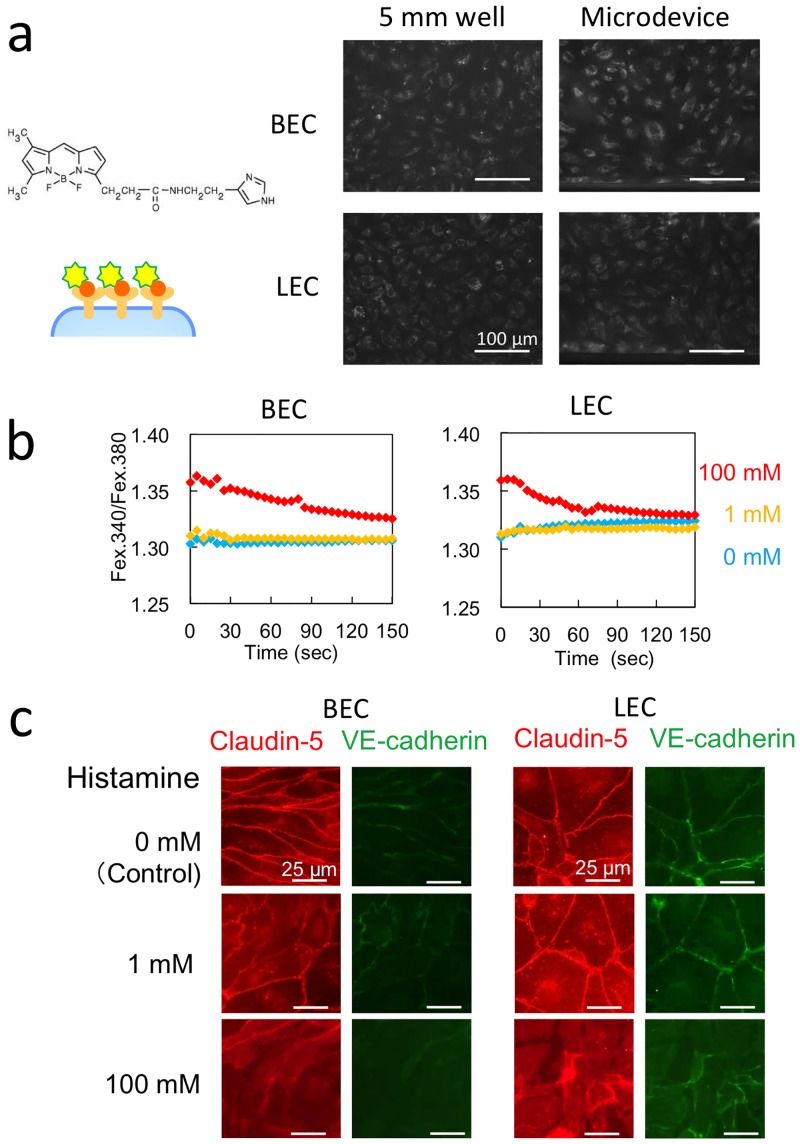
Effects of histamine stimulation on BECs and LECs cultured in the microfluidic system. A single-channel device without the permeable membrane was used instead of the coculture device. (a) Fluorescence imaging of BODIPY-FL-histamine bound to its receptor on BECs and LECs. (b) The time course of Ca^2+^ responses to histamine stimulation observed in BECs and LECs. (c) Immunostaining for claudin-5 and VE-cadherin after histamine stimulation for 30 min.

The binding of fluorescent-histamine to the BEC or LEC surface was examined using cells cultured in the single-channel device for 24 h under a 6-μL/h continuous-flow condition; the results are shown in Fig 5a. Under both the 6-μL/h continuous-flow condition generated using the syringe pump and the static condition in a 5-mm-diameter well, the binding of BODIPY-FL-histamine to its receptor was confirmed through fluorescence imaging. By contrast, no binding was detected in the case of BODIPY-FL-BSA (negative control; data not shown), which suggested that histamine binding was specific. Our results showed that histamine binding occurred in both microdevices under continuous-flow and conventional static conditions, and that histamine bound to both BECs and LECs.

Next, we measured intracellular Ca^2+^ levels. Whereas intracellular Ca^2+^ is maintained at a low level under quiescent conditions, histamine stimulation induces an increase in the level of intracellular Ca^2+^ by triggering the movement of Ca^2+^ from the lumen of the endoplasmic reticulum or the extracellular compartment into the cytosol through Ca^2+^ channels. To examine the behavior of intracellular Ca^2+^, we measured the fluorescence signal of the Ca^2+^ indicator Fura2 (which was loaded into the cytosol before the analysis) after adding the recording medium containing histamine-2HCl. The results of Ca^2+^ imaging are shown in Fig 5b. In both BECs and LECs, responses to histamine stimulation were detected at 100 mM. By contrast, response to stimulation with 1 mM histamine was detected in the well format, but no increase in fluorescence signals was observed in the microdevice, in which the Ca^2+^ level was already high before histamine stimulation. Intracellular Ca^2+^ was maintained at a higher level in the cells in the microdevice than in the cells in the 5-mm-diameter well (S3 Fig). Cells cultured in a microdevice were subject to strong shear stress during medium exchange, which might have resulted in higher Ca^2+^ levels.

To examine the effects of histamine stimulation on cell-cell junctions, medium containing 1 or 100 mM histamine was introduced into the microchannel at 6 μL/h by using the syringe pump, and after stimulation for 30 min, immunofluorescence staining for VE-cadherin and claudin-5 was performed. VE-cadherin and claudin-5 were clearly detected at cell-cell junctions in both BECs and LECs in the absence of histamine treatment (control), but the localization of VE-cadherin and claudin-5 changed when the histamine concentration was increased, and then the cell-cell junctions were gradually disrupted (Fig 5c).

We also performed immunofluorescence staining for VE-cadherin and claudin-5 after histamine stimulation of cells cultured in the 5-mm-diameter wells. Both BECs and LECs cultured in these wells showed a response to histamine stimulation that was distinct from that observed in the case of cells cultured in the microdevice (S4 Fig). The expression of VE-cadherin and claudin-5 was lower and localized in the cytosol in the cells cultured in wells when compared with the expression in the cells cultured in the microdevice. Stimulation with 100 mM histamine completely disrupted cell-cell contacts in both BECs and LECs cultured in wells. These results indicated that VE-cadherin and claudin-5 were not prominently localized at cell-cell junctions in cells cultured in wells, and thus cell-cell contacts were readily disrupted following histamine stimulation.

The aforementioned results agreed with those shown in Fig 4. Permeation levels were higher in the static culture than in the flow cultures. The responses to histamine stimulation differed between the well format and the microdevice because of the differences in cell-cell junction stability, which was induced under flow-culture conditions. The effect produced by the size of the culture area might not be related to the stability of cell-cell junctions.

### Permeability change induced by chemical stimulation

**Fig 6:**
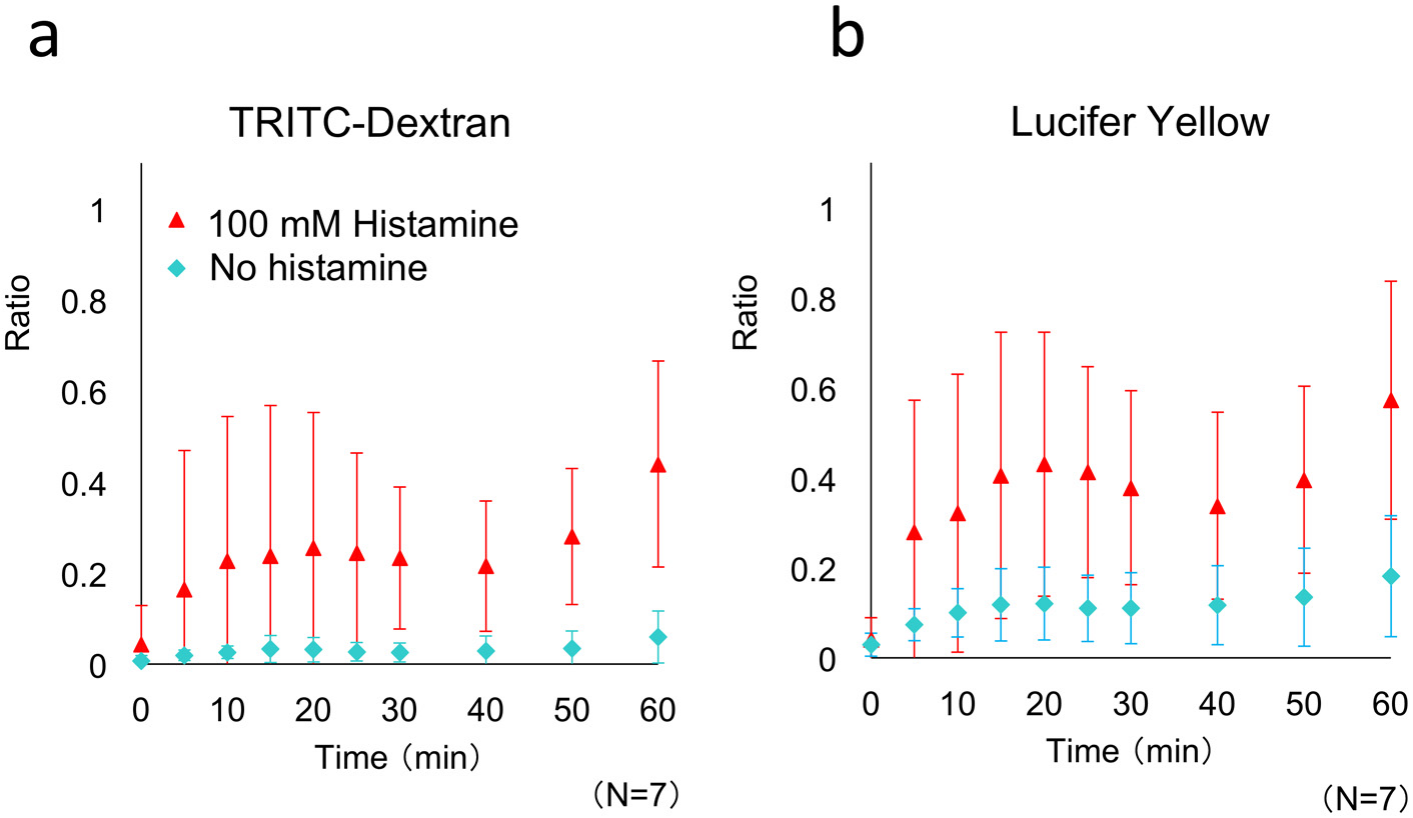
Permeability of BECs and LECs measured using the microfluidic system, with or without histamine stimulation. Permeability tests were performed after a 24-h cell culture (BEC-LEC coculture). Histamine (100 mM, red triangles) and control (blue diamonds). The values are presented as means ± SD. The fluorescent tracers used were (a) TRITC-dextran and (b) Lucifer Yellow. The results of *t* tests showed that the values obtained at 60 min with and without histamine stimulation were significantly different: (a) *P* < 0.001 and (b) *P* < 0.01. Each data point was obtained from seven devices (n = 7).

Because histamine stimulation affected cell-cell junctions in both BECs and LECs cultured in the microdevice, permeability tests were performed to examine the functions of the cells. In these tests, 100 mM histamine and the fluorescent tracers TRITC-dextran and LY (10 μM each) were added to the culture medium that was introduced into the upper channel (BEC channel) by using the syringe pump. The lower channel (LEC channel) was filled with culture medium containing 100 mM histamine. Fig 6 shows the permeability-test results, which are averages obtained from 7 experiments. Treatment with 100 mM histamine increased permeability in both BECs and LECs. The permeability coefficient calculated for 40-kDa TRITC-dextran was 6.4 × 10^−6^ cm/s, which is close to the value reported previously for a microfluidic microvessel model (4.8 × 10^−6^ cm/s for 20-kDa FITC-dextran) [46]. The results confirmed that histamine stimulation induced an increase in vascular permeability and a decrease in lymphatic absorption activity in the microdevice. This permeability test is relevant to inflammation caused by allergic reactions or bacterial infections, and showed, for the first time, that histamine impairs the function of cultured LEC as well as BEC by altering their intercellular permeability. Therefore, our proposed device may be applicable to the evaluation of anti-inflammatory agents, and for the screening of drugs such as antihistamines based on microcirculation involving LEC.

Next, the permeability test was conducted using habu snake venom. In the human body, a bite by this snake causes organ damage, because habu snake venom induces hemorrhage, myonecrosis, and edema. The most-accepted therapy for snake-bite patients is immediate administration of an anti-venom antiserum, which neutralizes the toxic activities of snake venom. The anti-hemorrhagic potency test is a major test used for measuring anti-venom activity, and in this test, rabbits are used [50]:}. The venom and the diluted anti-venoms are intracutaneously injected into depilated skin on the back of rabbits, and 24 h later, the rabbits are sacrificed (under anesthesia) and the test skin areas are immediately removed and placed on a glass plate in order to prevent distortion of their original shape. The diameter of each hemorrhagic spot is measured from the visceral side of the skin through the glass plate, the mean value is calculated, and the results are statistically analyzed. Based on animal-welfare concerns, the WHO recommend the development of alternative methods to animal testing in the preclinical evaluation of antivenoms (WHO Guidelines for the Production, Control and Regulation of Snake Antivenom Immunoglobulins, World Health Organization 2010)[51]. Therefore, we examined whether our microdevice can potentially be adapted for use in the anti-hemorrhagic potency test; we used habu snake venom and performed the vascular permeability test instead of performing the conventional hemorrhagic-spot analysis.

**Fig 7:**
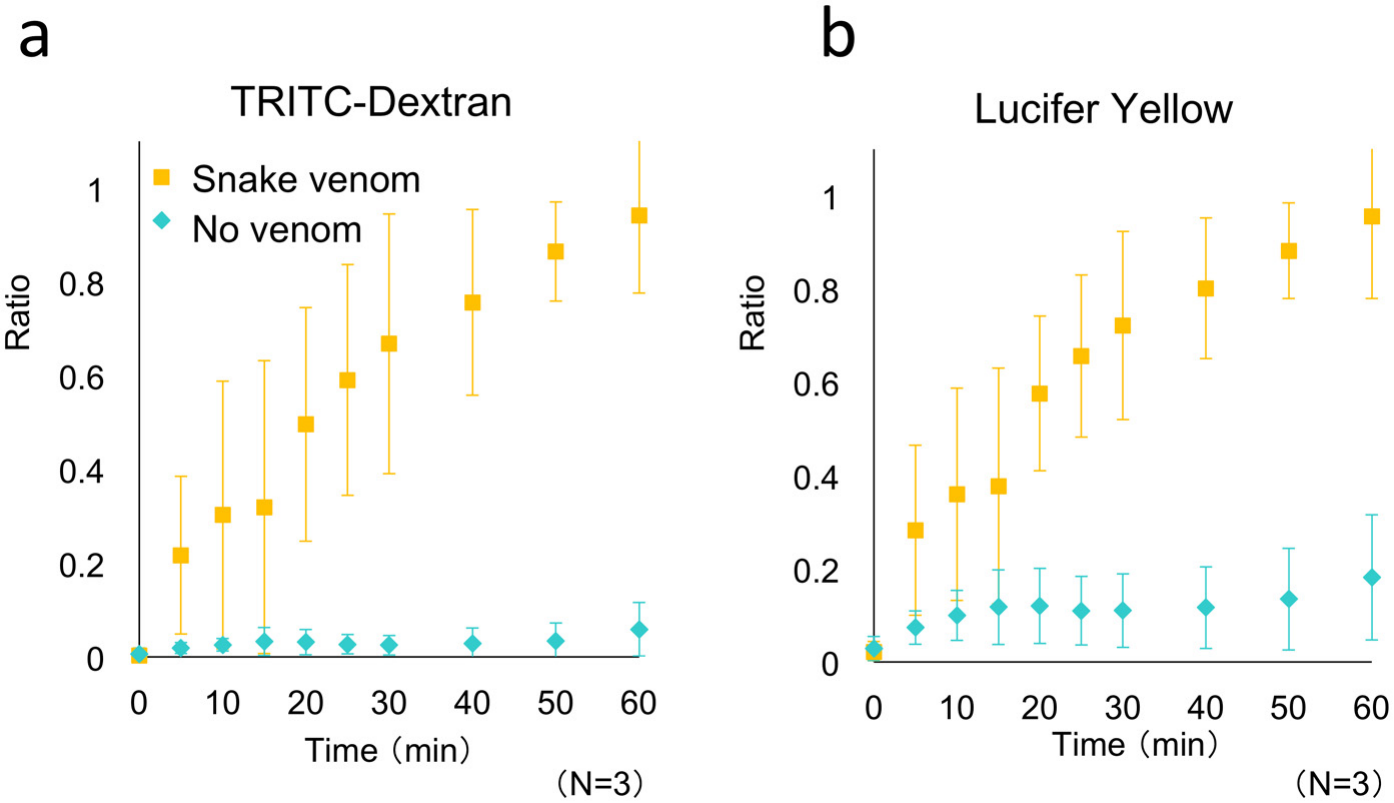
Permeability of BECs and LECs stimulated with or without habu snake venom. Permeability tests were performed after a 24-h cell culture (BEC-LEC coculture). Samples: (yellow squares) and control (blue diamonds). The values are presented as means ± SD. The fluorescent tracers used were (a) TRITC-dextran and (b) Lucifer Yellow. The *t*-test results showed that the 60-min values obtained without (control) and with stimulation with habu snake venom were significantly different: *P* < 0.0001, for both (a) and (b). Each data point was obtained from three devices (n = 3).

Permeation tests were conducted using the same format as that used for the histamine assay, but the test sample was now habu snake venom. Fig 7 shows the permeability-test results, which are averages obtained from three experiments. Permeability was increased in both BECs and LECs treated with 29 MHD/mL habu snake venom HR-2. These results confirmed that stimulation with habu snake venom induced an increase in vascular permeability in our microdevice. This permeability test with further improvements may be applicable to evaluation of antivenom and antidotes of snake venoms activity to neutralize toxicity in future.

Compared to histamine stimulation (Fig 6), treatment with habu snake venom induced a larger permeability change and elicited a more rapid response (Fig 7). This finding suggested that histamine and habu snake venom alter vascular permeability through distinct mechanisms. Histamine stimulation increased permeability by disrupting cell-cell contacts by inducing a change in the localization of proteins associated with tight and adherens junctions. The induced intercellular gaps were small and the opening and closing of the gaps were reversible. By contrast, habu snake venom increased the permeability because the protease present in the venom digested the ECM surrounding the cells, and this, in turn, induced the detachment of cells and generated wide gaps in the cell monolayer (S5 Fig). The response time measured for histamine stimulation suggests that the permeability increase involves multiple signal transduction steps that begin with histamine binding to its receptor and end with the alteration of the localization of proteins associated with tight and adherens junctions [47]. Conversely, stimulation with habu snake venom induced the permeability increase through protease cleavage-mediated cell detachment, which is a direct, one-step process, and thus the leakage of fluorescent tracers appeared within a few minutes.

In a previous study, BODIPY-FL-labeled casein was used in a habu snake venom activity test conducted without either animals or cells [52]. In this method, proteolytic cleavage-mediated increase in the fluorescence quenching of BODIPY-FL-labeled casein was measured [53]. The assay yielded results in a shorter time (~1 h) than did the anti-hemorrhagic potency test performed using rabbits. However, the results of the test based on proteolytic-cleavage activity were reported to not necessarily correspond to those of the hemorrhagic-activity tests [52], which indicates that hemorrhagic activity cannot be predicted based only on proteolytic-cleavage assays performed using isolated proteins. By contrast, the vascular-permeation assays conducted using our microdevice containing human BECs and LECs are based directly on vascular physiological behavior. This method reduces the assay time from the 24 h required for the conventional rabbit assay to 30 min, and it can be effectively used as a substitute for the anti-hemorrhagic potency test conducted using rabbits.

## Conclusions

A microcirculation model including both blood and lymphatic vessels was developed for examining vascular permeability. Claudin-5 and VE-cadherin were detected at cell-cell junctions in both BECs and LECs. The permeability coefficient measured here was lower than the value reported for isolated mammalian venules, which suggests that the BECs and LECs cultured in the microdevice retained their barrier functions due to the formation of tight endothelial junctions. Moreover, our results showed that flow cultures promoted the formation of endothelial cell-cell junctions. Histamine, an inflammation-promoting substance, induced changes in the localization of proteins associated with tight and adherens junctions and an increase in vascular permeability in the microdevice. These results indicate that both BECs and LECs appear to maintain their functions in the microfluidic coculture platform. The vascular damage induced by habu snake venom was successfully assayed using the microcirculation device, and the assay time was reduced from 24 h to 30 min.

This is the first report of a microcirculation model in which BECs and LECs were cocultured, and this model can potentially promote microcirculation research. Because the micromodel to realize application of this bioassay includes lymphatic vessels in addition to blood vessels, both vascular permeability and lymphatic absorption can be evaluated using this model. Therefore, the device could be used for research on immune surveillance, lymphedema, inflammation, and tumor metastasis, and could potentially also be used in the development of anti-inflammatory drugs as well as anticancer agents. Studies on drug-delivery systems (DDSs) have previously investigated the enhanced permeation and retention effect, which is caused by the presence of imperfect tumor blood vessels and poor lymphatic drainage in tumor tissues [54,55], and a lymphatic system-targeted DDS has also attracted research attention [56]. Because intractable diseases related to microcirculation exist, we expect the newly developed micromodel to make notable contributions by facilitating research on these diseases.

## Supporting Information

S1 Fig Microfluidic conditions used for cell culture. (TIF)

S2 Fig Typical fluorescence micrographs obtained in permeation tests. The bifurcation of the microchannels is shown. Red: upper channel. Green: lower channel.
(TIF)

S3 Fig Time course of Ca^2+^ responses to histamine stimulation observed in (a) BECs and (b) LECs cultured in the microdevice (single-channel device without the permeable membrane) or the 5-mm-diameter well for monoculture. (TIF)

S4 Fig Immunofluorescence staining for VE-cadherin and claudin-5 after histamine stimulation (the 5-mm-diameter well for monoculture). (TIF)

S5 Fig Typical cell morphology at 20 min after the addition of 1000-fold-diluted habu snake venom. The arrow indicates a gap in the cell layer.
(TIF)
