# Supplementary figures and images for: Microcirculation-on-a-Chip: A Microfluidic Platform for Assaying Blood- and Lymphatic-Vessel Permeability
Source: PLoS One. 2015 Sep 2;10(9):e0137301. doi: 10.1371/journal.pone.0137301 (PMC4558006; doi:10.1371/journal.pone.0137301)

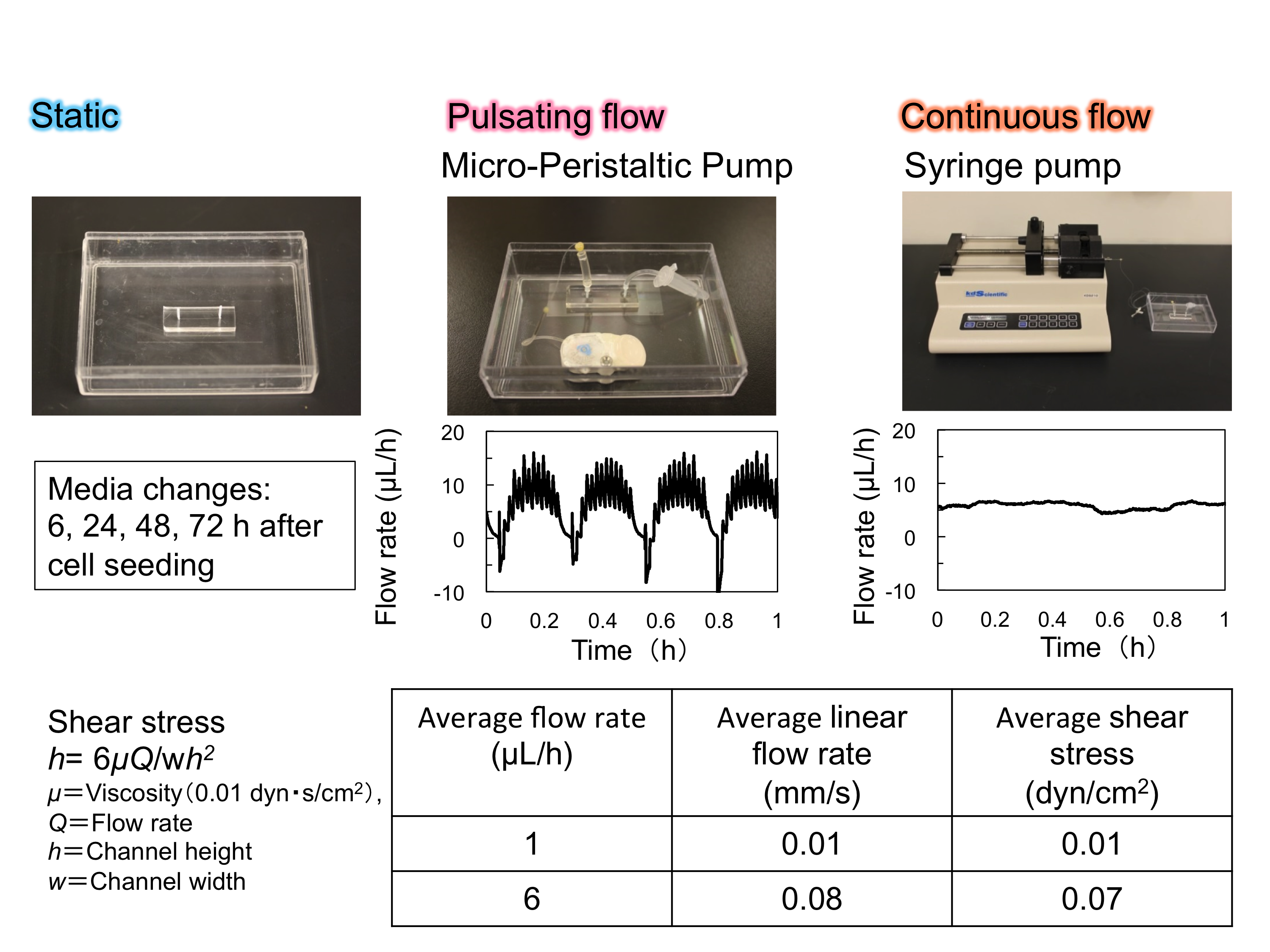

Supplement: S1 Fig — (TIF) [file pone.0137301.s001.tif]

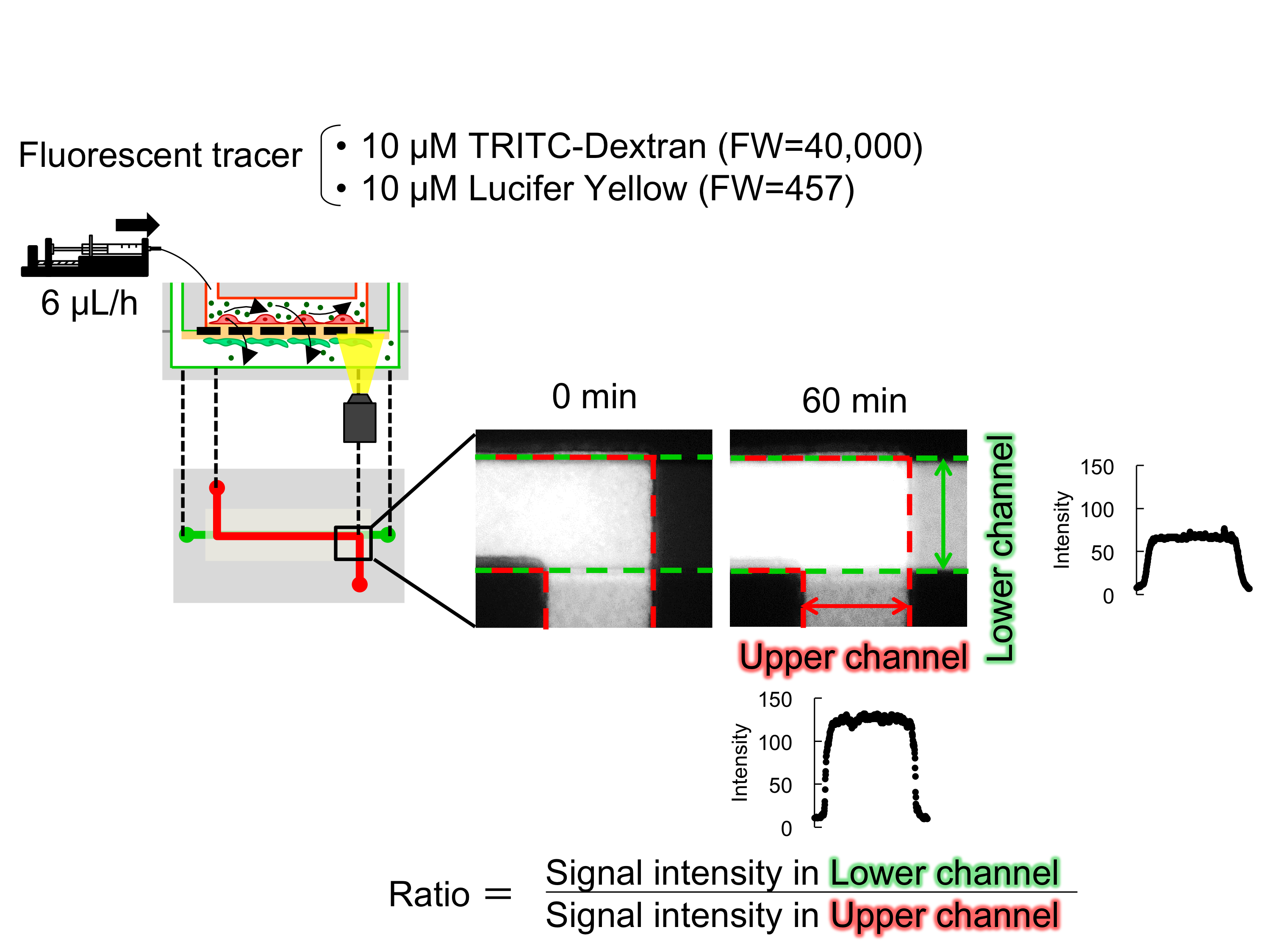

Supplement: S2 Fig — The bifurcation of the microchannels is shown. Red: upper channel. Green: lower channel. (TIF) [file pone.0137301.s002.tif]

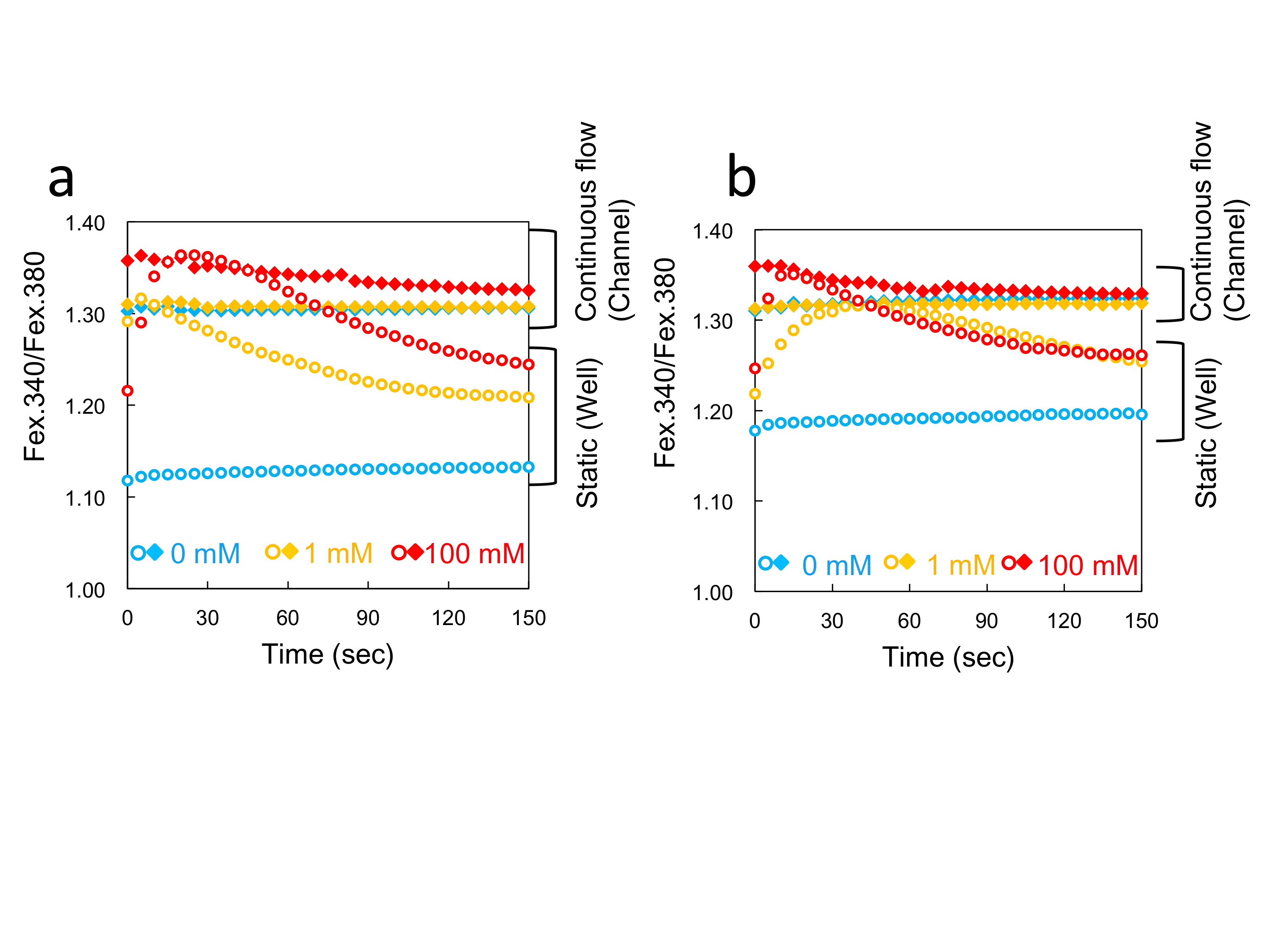

Supplement: S3 Fig — (TIF) [file pone.0137301.s003.tif]

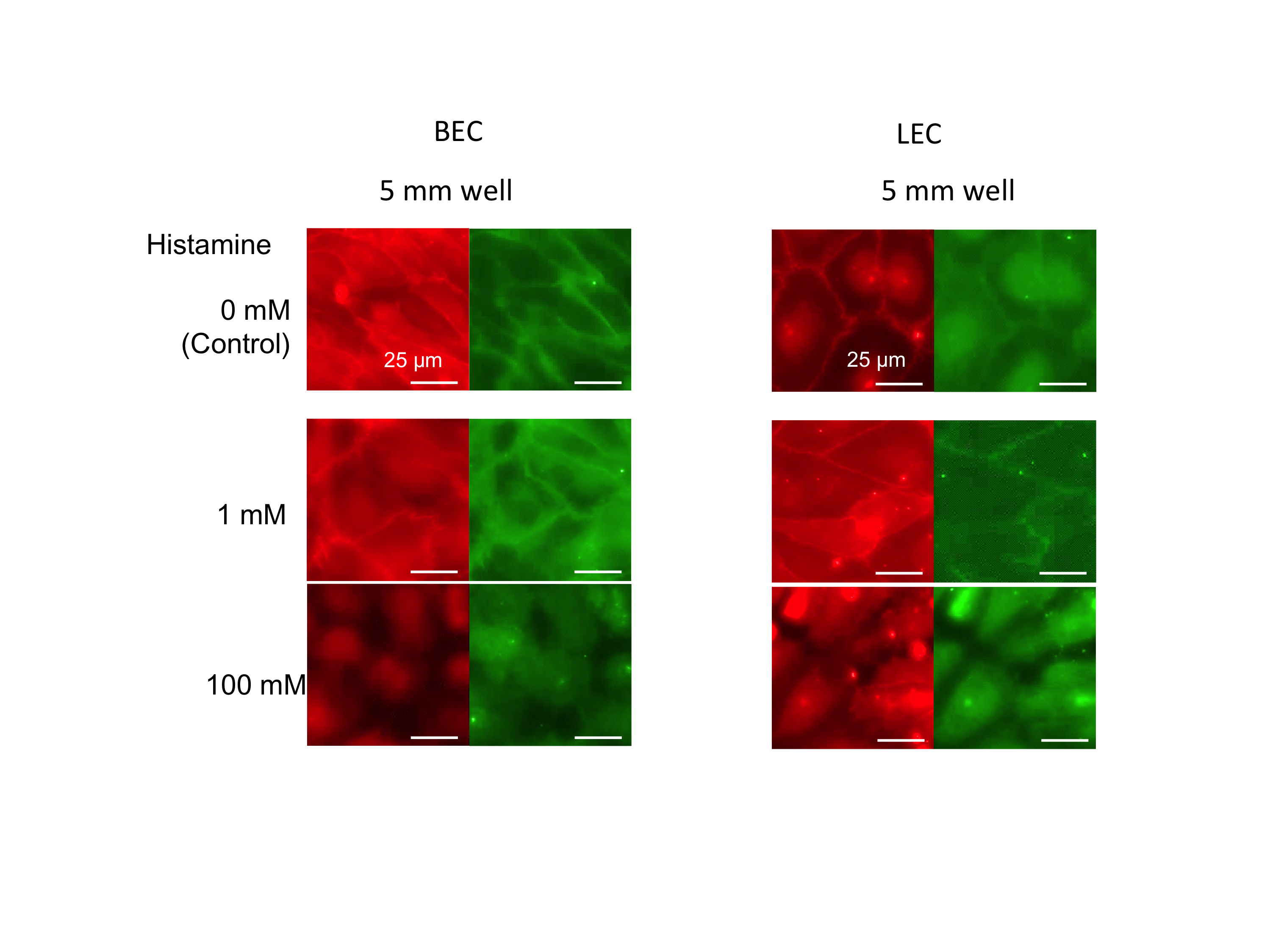

Supplement: S4 Fig — (TIF) [file pone.0137301.s004.tif]

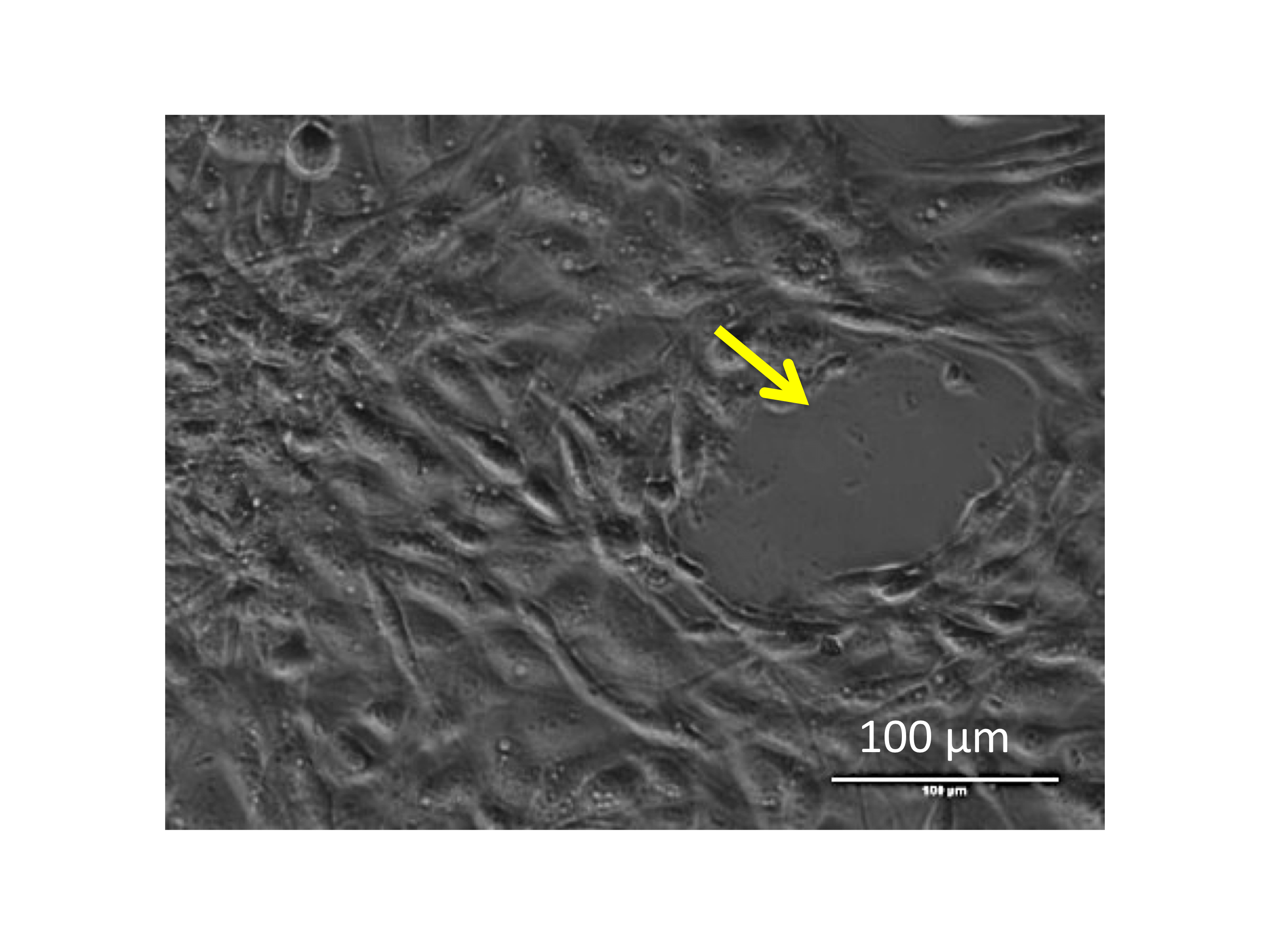

Supplement: S5 Fig — The arrow indicates a gap in the cell layer. (TIF) [file pone.0137301.s005.tif]
